# Our Bureau of Information

**Published:** 1911-11-11

**Authors:** 


### OUR BUREAU OF INFORMATION.

### Some New Developments.

This Bureau promises to be widely popular and useful. We have an increasing number of letters each week, and still further to aid in its organisation we call special attention to the following practical details :—

### Wanted, Experienced Correspondents.

To attain its maximum utility it is essential we should have the co-operation of correspondents up and down the country on whom we may depend for the good offices of making inquiries in regard to the accommodation offered, and to the circumstances of particular cases resident in any district they may kindly undertake. The work involved will not be necessarily arduous or continuous nor probably frequent, but the good which may be effected cannot fail to be appreciable. We want, for instance, this week a correspondent at Oxford, at Ledbury (Herefordshire), at Southbourne (Hants), at Bedford, Felixstowe, Malvern Link (Worcestershire), Norwich, Clacton, Maidenhead, and at Seaford (Sussex). It is essential that each correspondent shall have had some experience of and be interested in the alleviation of real suffering and misfortune. Each correspondent must conscientiously undertake to do the personal service required to the very best of his or her ability without delay, and we should prefer to find someone in each locality who has had experience as a district nurse, an almoner, a district visitor, a matron, secretary, or honorary secretary, or in some capacity where judgment and sympathy in handling such cases are essential to success. No expense will be entailed upon the correspondent, and all we ask for is whole-hearted co-operation and personal service. Applications accompanied by a statement of past experience and one or more good references should be sent to the Editor of The Hospital, 29 Southampton Street, Strand, London, W.C., and marked "Bureau of Information" in the left-hand top corner of the envelope.

### Offers of Accommodation.

Every letter relating to this department, or to any case mentioned in it, must be accompanied by a coupon as provided in Rule 5, and also by copies of two good references or testimonials. We have this week received four offers of accommodation, not one of which is accompanied by a coupon or any references. They are accordingly disqualified and unavailable.

### Offers of Help.

All offers of this kind must be accompanied in every case by a coupon as provided in Rule 5. We have received some excellent replies and offers of help in the cases published last week, for which we are most grateful.

### How to Make the Bureau a Success.

In order to facilitate as far as possible the work we have in view, everyone who uses or wishes to help our Bureau of Information must be kind enough to observe the following rules :

(1) Postal replies cannot be given. Exception will only be made in very special cases at the Editor's discretion.

(2) Queries or answers relating to different cases must be written, or preferably typed, on separate sheets of paper, and the writing must never be crossed.

(3) Questions and replies received not later than Wednesday will, as far as possible, be dealt with in our issue of the following Saturday week.

(4) Letters must have attached the name and address of the sender, with a pseudonym for publication if preferred.

(5) *All communications* for this department *must be accompanied by the coupon* to be found at the bottom of the third page of the cover on the inside, and should be addressed to the Editor of the Bureau of Information, c/o The Hospital, 29 Southampton Street, Strand, London, W.C.

*Notice*.—We invite workers of special experience and knowledge to offer help in replying to questions relating to their special department.

### NEEDS AND HELPERS.

### (1) INSTITUTION FOR INCURABLE CONSUMPTION.

"Kent" writes : I am urgently in need of information as to the form of application and conditions of admission to such an institution for one such sufferer, a woman. I want to have the addresses of one or more of these institutions, and I need all the help you can give to secure for her the best surroundings to cheer and help her to the end.

"White Cottage" suggests : The Mildmay Consumptive Home, Smyrna, Bronshill Road, Torquay, or St. Catherine's Home, Ventnor, might meet the case. They are both small homes for advanced cases. The payment is 10s. 6d. weekly at either. It is very difficult to find a home for incurables, as the object at most sanatoria is to provide for the hopeful cases that a cure may be effected if possible. As to the method of application, it is best to ask the medical man in charge of the case to make inquiries and explain the condition of the patient.

"Winchelsea" writes : Nearly all homes for consumptives have now discontinued the practice of taking incurable patients. The Firs Home, Trinity Road, Bournemouth, however, still receives advanced cases of this disease; payment 10s. 6d. a week. At Friedenheim, Upper Avenue Road, Swiss Cottage, they might receive such a case. Payment varies according to the room, and there are free beds. Acute and incurable cases are also received at St. Peter's Home, Mortimer Road, Kilburn, N.W., where payment is from 10s. to 21s. a week. There is also the "Hostel of God," 29 North Side, Clapham Common, under the East Grinstead Sisters, and here those in the last stages of consumption are sometimes received. The beds are free. In every case write to the Lady Superintendent, stating full particulars and asking for a form of application.

### (2) HOME WANTED FOR PARALYTIC MAN.

"Northampton" writes : Can you tell me of a suitable nursing Home for my father, who is paralysed? He has now been in that condition for about sixteen months, and has been steadily improving all that time with massage and exercise on the Sandow system, but his speech (the stroke affected the right side) does not seem to make the improvement I could wish. He is quite able to look after himself so far as dressing and undressing is concerned, so that he requires very little attention. I cannot pay a great deal—at the outside £2 per week—so perhaps you will answer accordingly. Is there any difficulty in getting patients into these Homes?

Nurse Dora, Arthur D. Smith, A. Simmons, T. Inglis, Nurse Channell, M. Spanton, M. Rideout, and F. Hooper are requested to send copies of their certificates and of not more than three testimonials.

### (3) HOME WANTED FOR DEFECTIVE BOY.

"Late Hospital Matron" writes : Can you tell me of a Home, near Worcester preferred, where a boy of nine years could be received for about £1 1s. per week. He has not walked or talked since birth, and cannot sit up without support.

E. L. Laxton and M. Spanton must send a coupon and also copies of their certificates and three testimonials.

### (4) INSURANCE OF PATIENTS.

The Superioress of Mercy Hospital is requested to send particulars of the information she needs with details and to enclose a coupon.

